# Combined treatment of dexamethasone mouthwash and low‐level laser therapy in the management of aphthous‐like ulcers caused by nonsteroidal anti‐inflammatory drugs: A case report

**DOI:** 10.1002/ccr3.8723

**Published:** 2024-03-27

**Authors:** Alaa ALhomsi, Abeer A. Aljoujou, Ammar Mashlah, Sanaa Al Ahdab, Haya Al Jabban

**Affiliations:** ^1^ Oral Medicine Department, Faculty of Dentistry Damascus University Damascus Syria; ^2^ Pharmacology and Toxicology Department, Faculty of Pharmacy Al Baath University Homs Syria; ^3^ Oral and Maxillofacial Surgery Department, Faculty of Dentistry Damascus University Damascus Syria

**Keywords:** adverse drug reactions, aphthous‐like ulcers, dexamethasone, low‐level laser therapy, nonsteroidal anti‐inflammatory drug

## Abstract

A case of major aphthous‐like ulcer was described in a 50‐year‐old patient. The patient showed the main signs of aphthous stomatitis painful ulcer, 1–2 cm in diameter, located on the ventral of the tongue, buccal mucosa, and the palate. These ulcers persisted for more than 3 weeks. The patient's self‐administration of a nonsteroidal anti‐inflammatory drug (NSAID) was suggested as the leading cause of aphthous‐like ulcers in this case. ulcers were treated with dexamethasone mouthwash and low‐level laser therapy (LLLT).

## 1 INTRODUCTION

Adverse drug reactions (ADRs) are defined as uncomfortable or harmful effects related to the use of a drug. ADRs may affect every part of the oral cavity and are the result of drugs taken either locally or systematically. They may have a variety of clinical manifestations; the most frequent are hyposalivation/xerostomia, burning mouth symptoms, ulcerative lesions, vesiculobullous lesions, red and white lesions, pigmented lesions, and dental anomalies.^1^ Adverse effects may be single or multiple lesions. Nonsteroidal anti‐inflammatory drugs (NSAIDs) are among the earliest drugs associated with ulcerative lesions in the oral cavity. Examples included piroxicam,^2^ naproxen,^3^ cyclooxygenase‐2 inhibitors,^4^ and ibuprofen.^5^ The diagnosis of adverse reactions in the oral cavity depends on the detailed medical history and clinical examination.^6^ An ulcer can be defined as a painful and well‐circumscribed depressed lesion associated with the absence of the superficial epidermal layer.^7^ Ulcers that resemble recurrent aphthous stomatitis but have systemic causes are often called Aphthous‐like Ulcers.^8^ The mechanism of the appearance of this ulceration is not clear. Small ulcers usually heal without scarring, while large ulcers heal with scarring.^6^ The presence of aphthous ulcers in the oral cavity negatively affects oral health and interferes with the quality of life‐related to oral health.^9^ Treatment of aphthous ulcers is challenging, as the causes remain unclear.^10^ Previous studies showed that the treatment begins with topical medications such as oral antiseptics, anti‐inflammatory agents, analgesics, antibiotics, local anesthetics, corticosteroids, and some natural substances.^11^ Laser has recently been applied in the aphthous ulcer treatment protocols,^12^ and many studies have focused on the effects of low‐level laser (light) therapy (LLLT) in treating aphthous ulcers. This type of treatment has been found to be safe with no side effects.^13^

## 2 CASE PRESENTATION

**FIGURE 1:**
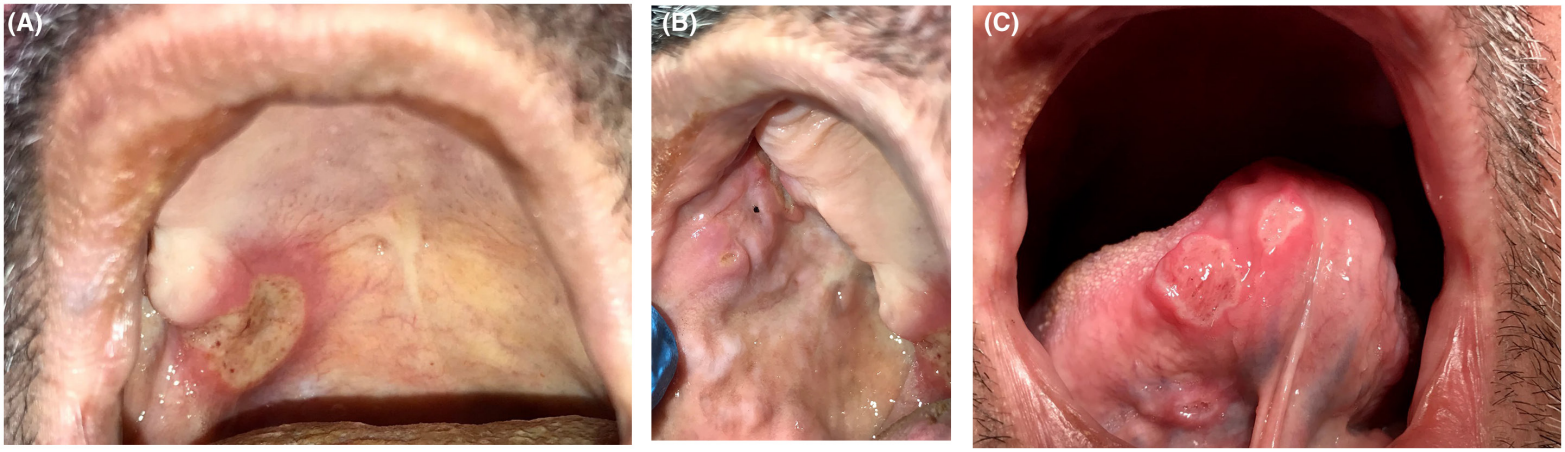
Ulcers on (A) the palate, (B) buccal mucosa, and (C) both ventral of the tongue.

A 50‐year‐old male was referred to the Department of Oral Medicine at the Faculty of Dentistry, Damascus University, with painful ulcers, 1–2 cm in diameter on the ventral of the tongue, buccal mucosa, and the palate (Figure 1). These ulcers were extremely painful and interfered with eating, drinking, and speaking. No history of smoking was reported. The patient stated that ulcers had appeared more than 3 weeks earlier and became bigger and more painful. We recorded his pain on a visual analog scale (VAS), with a score of 9. On clinical examination, there was a round‐shaped ulcer with erythematous borders. In the medical history, the patient stated that he had suffered from a sharp right earache for about a month, and he had been self‐medicating with an oral NSAID (50 mg of diclofenac potassium) six times a day. That is, the dose reached 300 mg per day for a month. Blood tests, serum vitamin levels, folic acid levels, iron, and ferritin were in the normal range (Table 1). Based on the presenting signs and symptoms, he was diagnosed as experiencing diclofenac‐induced aphthous‐like ulcers. The patient was referred to the otolaryngologist, and he was diagnosed with a middle ear infection. Subsequently, diclofenac was withdrawn and an antibiotic was prescribed. A written informed consent for the treatment and for the use of patient information was obtained, and the case was reviewed and approved by the Local Research Ethics Committee at Damascus University (No: 06202206).

**TABLE 1:** The patient's blood values.

Blood tests	Result
White blood cells (WBC)	8 × 10^3^/mm^3^
Red blood cells (RBC)	4.50 × 10^6^/mm^3^
Hemoglobin (HB)	13.20 g/dL
Hematocrit (Hct)	41.00%
Platelets (Plt)	300 × 10^3^/mm^3^
Vitamin B_12_	304.5 pg/mL
Hydroxyvitamin (D_2_ + D_3_)	58.76
Iron (serum)	80 mcg/dl
Ferritin	70 ng/mL
Folic acid	8.7 ng/mL

## 3 METHODS

Oral ulcers were managed with a mouthwash of dexamethasone 0.1 mg/mL q.i.d, for 5 days combined with a 980 nm diode laser treatment. A 300‐micron fiber was used, with a power setting of 0.8 W, continuous mode. The laser was applied without contact by maintaining a distance of 1 mm from the lesion and making continuous circular movements from the periphery toward the center of the ulcer. The application time was 30s for 3 days. The patient was followed up daily and evaluated for the general appearance of the lesion, pain reduction (VAS), and total healing time.

## 4 RESULTS

**FIGURE 2:**
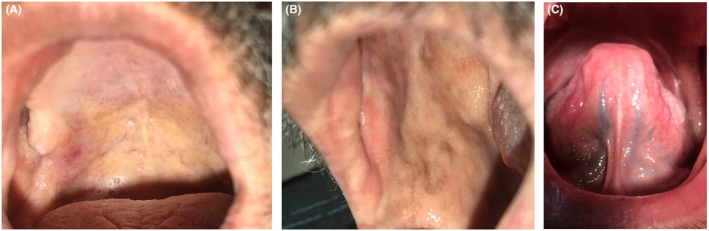
Ulcers healing after seven days and the combined treatment of a low‐level laser therapy and dexamethasone mouthwash. (A) on the palate, (B) buccal mucosa, and (C) both ventral of the tongue.

At the end of the first, second, and third treatment days, clear pain reduction (VAS) was recorded (VAS: 6, 5, and 2; respectively). The pain disappeared completely on the fourth day (Table 2). We recorded the day in which the lesions were re‐epithelized, and the final disappearance of the ulcers (Table 2). The ulcers were completely healed within 7 days and without any scars (Figure 2).

**TABLE 2:** Shows pain reduction (VAS) during the treatment days, and healing time of the ulcers.

VAS	Before the treatment	9
	First day	6
	Second day	5
	Third day	2
	Fourth day	0
	Fifth day	0
	Sixth day	0
	Seventh day	0
Healing time	Fifth day	The ulcers were re‐epithelized
	Seventh day	The ulcers were completely healed

## 5 DISCUSSION

Adverse drug reactions in the oral cavity are common with varied presentations and are sometimes difficult to diagnose. Diagnosis is usually based on the medical history and clinical findings.^14^ The severity of oral adverse reactions to drugs depends on the drug structure and composition, and duration of exposure to medication.^6^ After a few weeks of therapy, solitary oral ulcerations typically develop.^15^ Keeping the pill in the mouth for a while without swallowing it directly is the first culprit of oral ulcers. Ulceration can appear on every site of the oral mucosa, be painful, and with time become filled with granulation tissue.^1^ Although the exact molecular processes underlying these kinds of oral ulcerations are yet unknown, immune responses might be partially responsible.^3^ NSAIDs have the potential to harm the mucosa in several ways, such as topical irritation of the epithelium, mucosal barrier impairment, decreased mucosal blood flow, and disruption of the healing process.^16^ The main treatment aim for severe cases is to alleviate the pain, reduce ulcer size, shorten healing time, and allow healing without scarring.^17^ The patient received systemic antibiotics to treat the middle ear infection, but the function of microorganisms in the development and maintenance of chronic wounds, such as ulcers, is still not clear. Although it is assumed that all chronic wounds are contaminated with bacteria, it is still unclear when this contamination becomes an issue. There was no proof found for systemic antibiotics to be used in ulcer healing, indicating that there were no therapeutic advantages.^18^ Dexamethasone is a glucocorticoid commonly used as a topical treatment for aphthous stomatitis. Dexamethasone reduces inflammation by stopping the migration of white blood cells and reducing the capillary permeability, it is safe and effective in a randomized trial conducted on adults. A significant reduction in ulcer size and pain levels was observed after treatment. The risks and side effects associated with dexamethasone use such as the risk of developing oral candidiasis can be mitigated by reducing the duration of treatment.^19^ Currently, the use of LLLT has broad scientific support in clinical and laboratory studies. It has been proposed that LLLT can reduce pain and stimulate the healing process by the following mechanisms:

1. It affects the mitochondria, causing an increase it affects the mitochondria, causing an increase in ATP production, which increases oxygen consumption at the cellular level and thus promotes tissue repair.
2. Stimulates the secretion of serotonin and endorphins that produce an analgesic effect.
3. It reduces the synthesis of prostaglandins that produce anti‐inflammatory effects.
4. Improve blood circulation in the skin and mucous membrane.
5. It causes hyperpolarization by decreasing neuronal membrane permeability to Na/K.

Reduces edema by increasing lymphatic flow. The main advantage of LLLT is that its effect is localized, and it does not result in any damage to neighboring structures. It also relieves pain immediately.^13^

De Souza et al investigated the effects of diode laser therapy on pain reduction (VAS) immediately versus topical corticosteroid therapy and discovered no statistically significant differences were observed in terms of later pain relief and wound healing.^20^ According to the study by Lara et al, Which was performed on laboratory rats, affirming that the rats given topical dexamethasone showed morphological traits that were consistent with either the proliferative phase, or the healing phase of mucositis.^21^ However, the study of Bayat et al, determined that the number of cells in the repair was not significantly affected by the laser.^22^ Therefore, to reduce response time to treatment, we used the combined treatment of dexamethasone and LLLT. That's where Ramos et al study, demonstrated that the combination of dexamethasone and LLLT was an effective alternative, as they were able to heal ulcers in just 5 days.^23^ Based on scientific evidence, we chose to associate the mouthwash of dexamethasone and LLLT. We were able to heal the ulcers within 7 days without scarring, during treatment; there was a gradual improvement in recovery, which led to an improvement in the patient's quality of life. It is important to note that this is the first report presenting a severe case of aphthous‐like ulcers caused by NSAID and the use of combined treatment of Dexamethasone mouthwash and LLLT.

## 6 CONCLUSION

Oral ulceration may significantly affect quality of life and their treatment is a challenge for dentists. Especially if symptoms and pain were severe. Topical corticosteroids are the backbone of the treatment of aphthous‐like ulcers. The low‐level laser also reduces pain and speeds up recovery. Therefore, to improve response time and alleviate the condition we used combined treatment. Combining dexamethasone mouthwash, and applying a low‐level laser was a successful option.

The combined treatment of dexamethasone and laser appears to be promising. We suggest conducting clinical studies to confirm the achieved result.

## AUTHOR CONTRIBUTIONS

**alaa ALhomsi:** Conceptualization; data curation; investigation; methodology; project administration; resources; visualization; writing – original draft. **Abeer A. Aljoujou:** Conceptualization; data curation; investigation; methodology; resources; supervision; validation; visualization; writing – original draft. **Ammar Mashlah:** Data curation; formal analysis; investigation; methodology; visualization. **Sanaa Al Ahdab:** Formal analysis; investigation; methodology; supervision; validation. **haya Al Jabban:** Methodology; resources; writing – original draft; writing – review and editing.

## FUNDING INFORMATION

The authors have no sources of funding.

## CONFLICT OF INTEREST STATEMENT

The authors have no conflicts of interest to declare.

## ETHICS STATEMENT

Ethical approval was obtained from ethical review committee of Damascus University.

## CONSENT

A written informed consent was obtained from the patient involved in this study to publish clinical details and photographs.

## Data Availability

The data that support the findings of this study are available from the corresponding author upon reasonable request.
